# Dish layouts analysis method for concentrative solar power plant

**DOI:** 10.1186/s40064-016-3540-3

**Published:** 2016-10-22

**Authors:** Jinshan Xu, Shaocong Gan, Song Li, Zhongyuan Ruan, Shengyong Chen, Yong Wang, Changgui Gui, Bin Wan

**Affiliations:** 1 College of Computer Science, Zhejiang University of Technology, Hangzhou, 310013 People’s Republic of China; 2 College of Computer Science, Tianjin University of Technology, Tianjing, 300384 People’s Republic of China; 3 Business School, University of Shanghai for Science and Technology, Shanghai, 200093 People’s Republic of China; 4 Solarversal Technology, Hangzhou, 310021 People’s Republic of China

**Keywords:** Dish layout, Optimization, Shade calculation, Concentrative solar system

## Abstract

Designs leading to maximize the use of sun radiation of a given reflective area without increasing the expense on investment are important to solar power plants construction. We here provide a method that allows one to compute shade area at any given time as well as the total shading effect of a day. By establishing a local coordinate system with the origin at the apex of a parabolic dish and *z*-axis pointing to the sun, neighboring dishes only with $z>0$ would shade onto the dish when in tracking mode. This procedure reduces the required computational resources, simplifies the calculation and allows a quick search for the optimum layout by considering all aspects leading to optimized arrangement: aspect ratio, shifting and rotation. Computer simulations done with information on dish Stirling system as well as DNI data released from NREL, show that regular-spacing is not an optimal layout, shifting and rotating column by certain amount can bring more benefits.

## Background

The environment concerns caused by fossil fuel have promoted the development of clean and renewable energies. Solar energy with its unique properties becomes one of the most promising directions toward this goal, attracting research efforts from almost all areas (Razykov et al. 2011; Andraka et al. 2010; Wang et al. 2014). However, due to the relatively low energy flux density on earth ($\sim$1000 W/m$^2$), huge parabolic dishes are usually used to gather large area of sun radiation, in order to get desired output. For example, a 25 kW Stirling Solar Power System uses a parabolic dish with a total net reflective surface of $\sim$90 m$^2$ (Califonia 2001). Such huge parabola reflective mirror is hard to fabricate as a single unit. Typically small mirror facets are made, and assembled into parabola shape (dish alignment). Recently we developed a computer-based method allowing a quick and accurate alignment of facets (Wang et al. 2014), which ensures a safe and efficient operation of a solar dish Stirling system.

The efficiency of a single dish system can not guarantee the profit of a solar power plant which typically contains tens or hundreds of these dishes. For example, demonstration plants established or planning to be established in Europe, Japan, Australia and USA all contain a set of dish units (Mza et al. 2010; Abbas et al. 2009). In these cases, dishes might get shaded by their neighbors, leading to a decrease of efficiency. Furthermore, when a dish is shaded, a huge temperature difference would appear, resulting in an un-balanced displacement of pistons inside the Stirling engine and eventually a fatal damage to the engine. Therefore, for a solar power plant composed of dozens of these dishes, each dish should be well separated: no shading would be casted onto it by neighboring dishes during daytime. This requires a big area of field, leading to an excessive expenditure on wiring, land, etc. Thus, from the point of optimum profit, a compromise must be made between the efficiency of dishes and the land cost for installation.

This problem has attracted research efforts from scientists. Osborn (1980) proposed a model to analyze shade interactions among surrounding dishes in a small field, and came out with some optimized layouts for these fields based on energy production. Osborn assumed that the power output of a dish unit was proportional to the illuminated area. However the shape of dish was not considered in this model, which was important in detailed studies. Real data indicate that the output is reduced more rapidly than that of the illuminated area, and that a system cannot operate at all once a certain level of shading is reached. Igo and Andraka (2007) released a software, which in particular takes into account the impact of dish-to-dish shading on the energy output and revenue streams, to analyze the annual and monthly system performance. This method also incorporates local ground slope over the field, as well as zigzag layout between the rows of dish systems. The tool addresses only one aspect of the problem. In real field design, all aspects potentially affecting the output must be combined, for example the land costs, variation of sun flux energy during daytime, etc.

This paper aims at providing a systematic method for optimizing dish arrangement for large solar power plant. To allow readers combine the shading effect to others, we focus on presenting a mathematical model on shading area calculation and ways to search the optimized dish layout in multi-dimensional parameter space. For the purpose of better presenting, the rest of the paper is organized as follow: in “Mathematical bases” section we detail the mathematical model; A step-by-step guide of the numerical implementation of this method and definition of some key parameters are then presented in “Numerical implementation” section. We then apply this method to search for the optimized dish layout in “Application” section. A conclusion and short discussion of our model are presented in “Conclusion and discussion” section.

## Mathematical bases

**Fig. 1:**
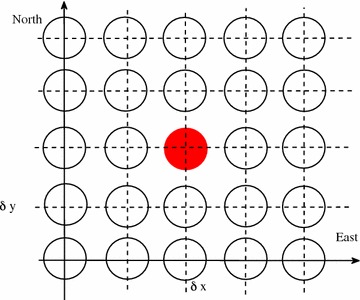
Schematic layout of solar power plant. Dishes are aligned in column-row mode with a distance of $\delta x$ in West–East direction, and $\delta y$ in South–North direction. All the calculations below are done to the dish in the center (*red filled circle*)

Dish Stirling power plant is typically organized in column-row format. To well describe the dish layout, we establish a ground coordinate, with one of the dishes as the origin, *x*-axis towards the East and *y*-axis toward the North, as shown in Fig. 1. The distance between successive dishes along *x*-axis is denoted as $\delta x$, and that along *y*-axis is denoted as $\delta y$. A compact plant design would be the smallest intervals along *x*- and *y*-axis ($\delta x, \delta y$) that give no shading to any dish in the field.

The shade of each dish depends sensitively on the sun position, which is accurately modeled in Ref. Reda and Andreas (2004), Grena (2008) and the distance to its neighbors. For the dish located most East and South, no shade would be casted on to it in the morning, while would be shaded in the afternoon. For the dishes in the center, shade could appear at any time.

**Fig. 2:**
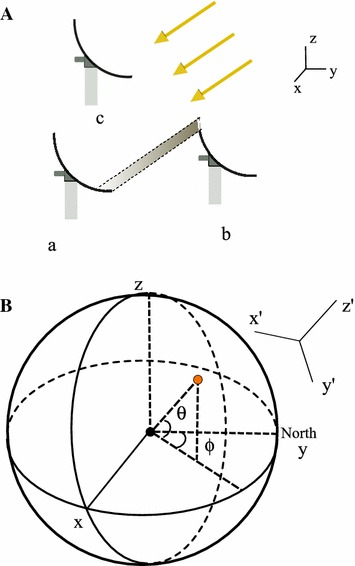
**A** Dishes in a plant field can be referred by a natural coordinate system with *x*-axis toward the East, *y*-axis toward the North and *z*-axis showing the altitude. The built-in sun tracking system of each dish ensures that all dishes face toward the sun. The *shade area* would only be the regions where dishes overlap along the projection of sun radiation. **B** A local (dish) coordinate ($x'-y'-z'$) with $z'$-axis pointing at the sun (*red-filled dot*, described by its azimuth angle $\phi$ and elevation angle $\theta$) is originated at the dish. Neighboring dishes shading to it would only be those with $z'$ values larger than 0

The solar Stirling power system has a sun tracking routine incorporated, ensuring each dish facing toward the sun. As a consequence, the potential shade on dishes would follow the shape of the dish, and only the dishes that lie in front of a given dish would cast shade onto it (see Fig. 2A).

With this concept in mind, we establish a dish (local) coordinate $x',y',z'$, with $z'$-axis pointing to the sun, shown in Fig. 2B. The sun position in the earth coordinate system is described by the azimuth angle $(\phi )$ and elevation angle $(\theta )$, its position in the dish coordinate system can be obtained by rotating around *z*-axis anti-clock wisely by $-\phi$ and then rotating around the *x*-axis anti-clock wisely by $-(\frac{\pi }{2}-\theta )$. Thus the rotation matrix that converts the earth coordinate *x*, *y*, *z* into dish coordinate $(x',y',z')$ is expressed as

1 $$\begin{aligned} \begin{aligned} {\mathbf {R}} &=R_{x}\left(-\left(\frac{\pi }{2}-\theta \right)\right) R_{z}(-\phi ) = \left( \begin{array}{ccc} \cos \phi &{}\quad \sin \phi &{}\quad 0\\ -\sin \theta \sin \phi &{}\quad \sin \theta \cos \phi &{}\quad \cos \theta \\ \cos \theta \sin \phi &{}\quad -\cos \theta \cos \phi &{}\quad \sin \theta \\ \end{array}\right) \end{aligned} \end{aligned}$$

For a particular dish ($D_0(x_0,y_0,z_0)$) in the field, its neighbor $D_i(x_i,y_i,z_i)$ can be expressed as $D'(X,Y,Z)=(x_i-x_0,y_i-y_0,z_i-z_0)$ relative to $D_0$, with $X_i = x_i-x_0, Y_i = y_i-y_0$ and $Z_i = z_i-z_0$. This can further be expressed in the dish coordinate system as

2 $$\begin{aligned} \begin{aligned} D_i(x'_i,y'_i,z'_i)&={\mathbf {R}} \left( \begin{array}{c} X_i\\ Y_i\\ Z_i\\ \end{array}\right) \end{aligned} \end{aligned}$$

Here $D_i(x'_i,y'_i,z'_i)$ represents the position in the dish local coordinate system $(x',y',z')$. As a consequence, the neighboring dishes that would potentially cast shade on to dish $D_0$ are those with $z'_i>0$.

**Fig. 3:**
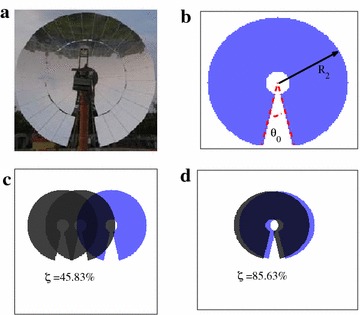
Parabolic dish layout. **a** Dish of 25 kW Stirling solar power unit installed at Solarversal Hangzhou site. To allow free tracking, a notch is made to the parabolic dish to avoid intervening between dish and the supporting pedestal. **b** Its net reflecting area can be model by a sector ring, which is described by Eq. 3. **c**, **d** Depending the sun positions, shades will be casted to the dish by its neighbors, reducing the effective reflecting area (*light blue area*)

The solar energy collecting device is composed of a set of mirror facets forming a parabola shape, which is ideal for collecting sun radiations parallel to the optical axis of the dish. To ensure a good performance, two strategies are applied: first, the facets are finely aligned with each light spots reflected from these facets evenly lying within the heat-head of the power conversion unit (PCU) of the dish Stirling system. Several methods have been developed to achieve a good alignment (Wang et al. 2014; Carlson et al. 2011). Second, the dish assembly incorporates 2-axis tracking system with an elevation and an azimuth drivers, which guarantees parallelism of the sun radiation with the optical axis. To allow free movements of the dish in tracking mode, a notch is made to the parabolic dish in order to avoid intervening between mirrors and supporting pedestal (Fig 3a). The projection of the dish along sun incident direction is a sector ring, as showing in Fig. 3b. Thus the effective area for collecting sun radiation

$S_R$ can be described as:

3 $$\begin{aligned} S_{R}(r,\theta )=\int _{R_1}^{R_2}\int _{\frac{\theta _0}{2}}^{2\pi -\frac{\theta _0}{2}}r\mathrm {d}r\mathrm {d}\theta \end{aligned}$$

Here $R_1=1027$ mm is the radius of the hub, $R_2=5700$ mm is radius of parabolic rim and $\theta _0$ is the notch angle in radian (Andraka et al. 2011).

Depending on the sun position and the distances to its neighbors, a dish might get shaded, leading to a significant reduction of effective reflective area. Figure 3c, d show this effective area (light blue region) varies with changes in dish distance $\delta x, \delta y$ and time. With the increase of shade area, extreme temperature unbalance on the heat-receiver plane of the engine builds up, and eventually leads to a significant reduction of plant efficiency. To avoid this, large distance among dishes is required, leading to an augmentation of investment costs. Here we target a mathematical description of shade, which is then to be combined with other effects to come out an optimized solar power field layout design.

A typical scheme of solar plants is shown in Fig. 1. Dishes (represented as circles) are arranged in column-row mode, corresponding to East–North. We label the distance between columns (East–West direction) as $\delta x$, while that between rows (South–North direction) as $\delta y$. One can easily figure out that dishes on the most-east column will not get shaded in the morning, while highly possible get shaded in the afternoon; dishes on the most-south row will not get shaded in the summer, while highly potential in the winter depending on the latitude of the field.

To make our analysis as general as possible, we first look at a dish in the center (red circle in Fig. 1). Its neighbors are dishes located at $(n\delta x, m\delta y, \delta z)$ in field coordinate system, with $n,m= \pm 1, \pm 2, \pm 3 \ldots , etc.$ We ask the question how the shade varies over time at given $\delta x$ and $\delta y$. We then consider the total shade effect with respect to total irradiation as a function of $\delta x$, $\delta y$ and $\delta z$, from which one would figure out the optimized distance among dishes $\delta x$ and $\delta y$.

## Numerical implementation

With the knowledge of complexity of theoretical analysis of the problem and the increasing computational power of modern computers, we choose to implement the analysis numerically. In this section, we detail the procedures of doing it.

### Expressing neighboring dishes in field coordinate system (*X*, *Y*, *Z*)

As dishes are arranged in row-column form, positions of neighboring dishes related to a specific dish $D_0$ we consider are expressed as

$$\begin{aligned} D_i(X,Y,Z) =(m\delta x, n\delta y, \delta z) \end{aligned}$$

with *m* being number of rows between *ith* dish $D_i$ to $D_0$, *n* being number of columns between $D_i$ to $D_0$, and $\delta z$ being the difference in altitude between $D_i$ and $D_0$.

### Expressing neighboring dishes in dish local coordinate system $(x',y',z')$

The dish local coordinate system $(x',y',z')$ is defined with its origin at the apex of $D_0$, and $z'$-axis pointing to the sun. At any time, we compute the sun position $(\theta ,\phi )$ using algorithms found in Ref. Reda and Andreas (2004). The rotating matrix that transforms the field coordinate system into dish coordinate system is then obtained using Eq. 1, with which positions of neighboring dishes can be calculated (see Eq. 2).

### Selecting suspicious dishes

Expressing neighboring dishes in the dish local coordinate system enables us excluding un-suspicious neighbors, i.e., dishes with $z'<0$. That is to say, neighboring dish $D_i (x'_i,y'_i,z'_i)$ only with $z'>0$ is possible for casting shade on $D_0$. As sun light is parallel to $z'$-axis, the shape of shading from a dish is identical to its projection along $z'$-axis.

### Finding out shaded region

**Fig. 4:**
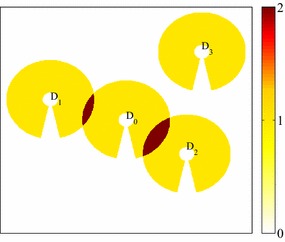
Numerical scheme for determining the shaded region on dish $D_0$. For each suspicious dish $D_i$ ($i =1,2,3$), its shade in the dish local coordinate system is drawn using Eq. 3 and labeled with a value 1, even for the points that are shaded by more than one suspicious dishes. The reflective region of dish $D_0$ is also drawn and marked with value 1. Adding to the entire shade from suspicious dishes yields the shaded area on $D_0$ with value 2 (*red region*)

To determine the amount of shaded region on $D_0$, we first draw out the shade of a suspicious dish $D_i$ using Eq. 3 in the dish local coordinate system, and label it with value 1, even for points that are shaded by more than one suspicious dishes. In this case, the total shade from suspicious dishes is then determined, as shown in Fig. 4. We then draw out dish $D_0$ in the same coordinate system, and label its reflecting region with 1. By adding these two together, the shade from neighboring suspicious dishes on dish $D_0$ is shown with a value of 2. As a consequence, the shade region $S_{shade}$ can be obtained simply by counting the number of points with value greater than 1. Thus the shading percentage at a given time with fixed $\delta x$ and $\delta y$ is defined

4 $$\begin{aligned} \zeta = \frac{S_{shade}}{S_R}\times 100\,\% \end{aligned}$$

**Fig. 5:**
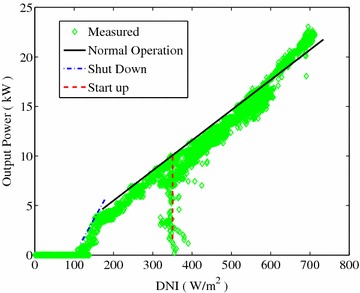
Measured output power of Dish Stirling Solar Power System at Solarversal Hangzhou site. To start up, DNI value up to 350 W/m$^2$ is required, while it shuts down with DNI close to 100 W/m$^2$

Solar thermal power plant is different from the PV panel, which generates electricity as long as the existence of solar irradiance. The Dish Stirling Solar Power system requires high sun radiation to start up, while can operate at low radiation due to its heat capacity. As shown in Fig. 5, the Stirling system requires DNI value larger than 350 W/m$^2$ to start up, while persists operating with the DNI close to 100 W/m$^2$. Considering the fact that shading most likely appear in the morning and afternoon, at which time DNI is too low to be used even without being blocked by neighbors, so the essential effect to the profits of a power unit can be expressed as

5 $$\begin{aligned} \zeta _e =\int _{D=1}^{D=365} \frac{\int _{t_r}^{t_s}\zeta \times \text {DNI}\cdot \text {d}t}{\int _{t_r}^{t_s}\text {DNI}\cdot \text {d}t}\cdot \text {d}D \times 100\,\% \end{aligned}$$

where $t_r$ and $t_s$ are the time at which DNI ($\ge$50 W/m$^2$) first reaches the required value in the morning and first moves out of the required range (DNI $\le$ 100 W/m$^2$) in the afternoon, respectively. This guarantees the exclusion of energy loss due to low intensity, and ensures it focuses only on the loss of sun energy due to shade.

## Application

The aim of establishing solar power plant is to pursuit high profits. As long as a location is selected, for a fixed plant capacity, optimum investment can only come from pipe/circuits and lands. Intuitively, the first two factors are directly related to land cover area. The more land used, the higher extra-investment, while less efficiency lost due to shading. Thus a good plant design should compromise between land cover ratio and output efficiency.

To set a ground for comparison, we fix the land cover ratio $\eta$, defined as the ratio between the total reflecting area of dishes and the total land they occupied, and vary the layout of a plant. Under this condition, an optimized design can be labeled by the lowest shading effect.

**Fig. 6:**
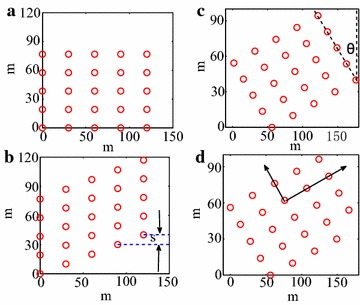
Representation of dish layout generated by introducing shifting *s* and rotating angle $\theta$ from the base layout, i.e., regular spacing lattice with column distance $\delta x$ pointing to the East, and row distance $\delta y$ pointing to the North (**a**). **b** Shift each column by *s*; **c** rotate anticlockwise by $\theta$. **d** In order to keep the land cover ratio constant, scaling parameter $\gamma$ is introduce to elongate the column and row distances

Solar power units are typically spaced regularly in a lattice, with row direction pointing to the East, and column pointing to the North. Recent work suggests that irregular spacing can improve efficiency (Cumpston and Pye 2014). To find the optimized layout design, we start with a regular spaced lattice ($\delta x, \delta y$), as shown in Fig. 6a, as a base. Other layout patterns can be obtained by shifting the column by *s* (see Fig. 6b) and rotating it by $\theta$ (see Fig. 6c). In order to set the land cover ratio unchanged, a scale factor $\gamma$ is introduced. With these mathematical representations, the search for optimized layout turns into a search in parameter space $\delta x,\delta y,\theta$ and *s* that gives the lowest shading effect.

Searching in 4-D space is not an easy task, we here break down the question into two parts: (1) search for optimized regular spacing layout (with $\theta =0,s=0$); (2) search in $\theta$ and *s* with optimized values of $\delta x$ and $\delta y$ obtained from previous step. In the case of regular spacing with fixed land cover ratio $\eta$, a dish layout can be expressed by a single parameter $\delta x$, due to the relationship:

6 $$\begin{aligned} \eta = \lim _{N\rightarrow \infty }\frac{N* S_{R}}{\text {Land Used}} = \frac{S_{R}}{\delta x\cdot \delta y} \end{aligned}$$

**Fig. 7:**
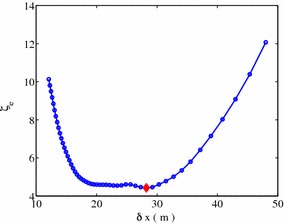
Essential shading effect under regular spacing layout with land cover ratio $\eta =0.1835$. $\delta x=28$ (*red diamond*) gives an optimized regular spacing layout. Calculation is done with DNI data of Delingha (longitude 97.37, latitude 37.37) released by NREL

Figure 7 shows the calculated essential shading effect under different column distance $\delta x$ with a fixed land cover ratio $\eta = 0.1835$. It is seen that with the increase of the distance along East–West direction $\delta x$, essential shading effect $\zeta _e$ decreases first, while increases later. This phenomena is intuitive, as shading mainly comes from the E-W direction at small $\delta x$ (large $\delta y$). With the increase of $\delta x$ (decrease of $\delta y$) shading on the N-S direction overwhelms, which causes the shading effect $\zeta _e$ to increase again. An optimized layout arises at $\delta x\approx 28$, with an annual effective shading effect $\zeta _e=4.431\,\%$.

A further optimization is done to the previously optimized regular-spacing layout by shifting each column by *s* and rotating columns anti-clock wisely by $\theta$. We numerically search for the minimum shading effect in this $(\theta ,s)$ 2-parameter space. To set a ground for comparison (fixed land cover ratio $\eta$), a scaling factor $\gamma$ is introduced to the base rectangle of width $\delta x$ and length $\delta y$ before shifting *s* and rotation $\theta$ are made. Setting the surface area of the resulted parallelogram after transforming equal to that of the original base rectangle $\delta x, \delta y$ comes:

7 $$\begin{aligned} \gamma = \frac{-s\sin {\theta }+\sqrt{s^2\sin ^2{\theta }+4\delta ^2 x \cos {\theta }}}{2\delta x\cos {\theta }} \end{aligned}$$

**Fig. 8:**
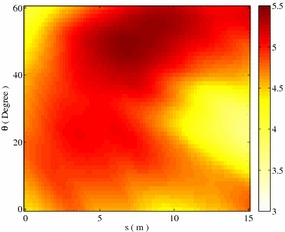
Calculated percentage of shading effect by rotating and shifting the base layout by $\theta$ and *s* respectively. An optimized layout appears at $s\approx 15, \theta \approx 22.5^\circ$,with $\zeta _e = 3.12\,\%$

Figure 8 gives the essential shading effect(in percentage) by shifting and rotating the base layout obtained previously. As it is shown, introducing shift *s* and rotating $\theta$ further reduces $\zeta _e$, leading to an global optimized layout under fixed land cover ratio and system specification. Comparing to regular-spacing layout, shading effect can be reduced up to 3.12 %, which is about 30 % smaller than the optimized regular-spacing layout, by shifting each column 15 m and rotating 22.5$^\circ$.

## Conclusion and discussion

Solar power plant as one of the most promising options for providing renewable clean energy has been attracting more and more interests. But there are still a set of questions need to be addressed. In this paper, we focused on the calculation of shade effect and its application on Dish Stirling Solar Power Plant design.

To maximize the profit of a solar power plant, a compromise should be made among factors that would potentially increase/decrease the costs. Shading as one of those factors is of crucial importance to the safety as well as profit of a solar power system: increasing distance among dishes reduces the shaded area, resulting an increase of power conversion efficiency, at the same time increases land costs and construction investment. In order to find an optimized layout among these controversial factors, we established a comparing ground by fixing land cover ratio. Under this condition, an optimized design can then be labeled by searching the lowest essential shading effect $\zeta _e$ in three dimensional parameter space $(\delta x, \theta , s)$.

To calculate the shading effect, we proposed a mathematical model that allows one to easily compute the shade area at a given time as well as total percentages of shade of a day, and can be easily extended to the analysis of total shade effect over a whole year. Considering the fact that solar power conversion unit may not take use of low sun radiation, which often accompanied by low sun elevation angle (high shading area), we combined this method with the specification of power conversion unit, and proposed the essential shading effect $\zeta _e$ as an ideal optimizing parameter.

Despite the computational power of modern computers, searching in 3-D space $(\delta x, \theta , s)$ is still not an easy task, especially computing all year long shading with desired precision. We here split the search procedure into two steps: (1) 1-D search for optimal regular-spacing layout; (2) 2-D search for global optimal layout. Taking into account of the properties of the Dish Stirling Energy System and DNI data, our results show that neither the frequently used regular-spacing nor Zig-zag shape is an optimal layout. The latter may even increase the shading effect, i.e., reducing efficiency (Igo and Andraka 2007). An optimum arrangement is obtained by shifting each column by 15 m and rotating by $22.5^\circ$.

Although the results presented in the paper are obtained at a particular location (longitude 97.37, latitude 37.37), the method behind is quite general, it can be repeated for other locations by using different DNI data and properties of different power conversion unit.
